# Design of a randomized controlled trial of disclosing genomic risk of coronary heart disease: the Myocardial Infarction Genes (MI-GENES) study

**DOI:** 10.1186/s12920-015-0122-0

**Published:** 2015-08-15

**Authors:** Iftikhar J. Kullo, Hayan Jouni, Janet E. Olson, Victor M. Montori, Kent R. Bailey

**Affiliations:** From the Division of Cardiovascular Diseases, Department of Medicine (IJK, HJ), Department of Health Sciences Research (JEO, KRB), Knowledge and Evaluation Research Unit (VMM), Mayo Clinic, 200 First Street SW, Rochester, MN 55905 USA

**Keywords:** Clinical trial, Design, Coronary heart disease, Disclosure, Genetics, Genetic risk, Genetic testing, Personalized medicine

## Abstract

**Background:**

Whether disclosure of a genetic risk score (GRS) for a common disease influences relevant clinical outcomes is unknown. We describe design of the Myocardial Infarction Genes (MI-GENES) Study, a randomized clinical trial to assess whether disclosing a GRS for coronary heart disease (CHD) leads to lowering of low-density lipoprotein cholesterol (LDL-C) levels.

**Methods and design:**

We performed an initial screening genotyping of 28 CHD susceptibility single-nucleotide polymorphisms (SNPs) that are not associated with blood pressure or lipid levels, in 1000 individuals from Olmsted County, Minnesota who were participants in the Mayo Clinic BioBank and met eligibility criteria. We calculated GRS based on 28 SNPs and will enroll 110 patients each in two CHD genomic risk categories: high (GRS ≥1.1), and average/low (GRS <1.1). The study coordinator will obtain informed consent for the study that includes placing genetic testing results in the electronic health record. Participants will undergo a blood draw and return 6-10 weeks later (Visit 2) once genotyping is completed and a GRS calculated. At this visit, patients will be randomized (1:1) to receive CHD risk estimates from a genetic counselor based on a conventional risk score (CRS) vs. GRS, followed by shared decision making with a physician regarding statin use. Three and six months following the disclosure of CHD risk, participants will return for measurement of fasting lipid levels and assessment of changes in dietary fat intake and physical activity levels. Psychosocial measures will be assessed at baseline and after disclosure of CHD risk.

**Discussion:**

The proposed trial will provide insights into the clinical utility of genetic testing for CHD risk assessment.

**Clinical trial registration:**

ClinicalTrials.gov registration number: NCT01936675.

**Electronic supplementary material:**

The online version of this article (doi:10.1186/s12920-015-0122-0) contains supplementary material, which is available to authorized users.

## Background

The potential use of genetic testing for estimating risk of common diseases is of great scientific and public health interest [1]. In one of the first genome-wide association studies (GWAS) [2], common genetic variants in the 9p21 region were associated with coronary heart disease (CHD) with an odds ratio of 1.22 per risk allele. Subsequent meta-analyses in largely European ancestry populations identified additional loci of modest effect sizes associated with CHD [3,4]. The majority of these loci are associated with CHD independent of conventional risk factors and could potentially improve the accuracy of CHD risk estimates. Indeed several studies have reported that a genetic risk score (GRS) based on multiple CHD susceptibility single-nucleotide polymorphisms (SNPs) provides incremental information for CHD risk estimation [5,6].

**Fig. 1:**
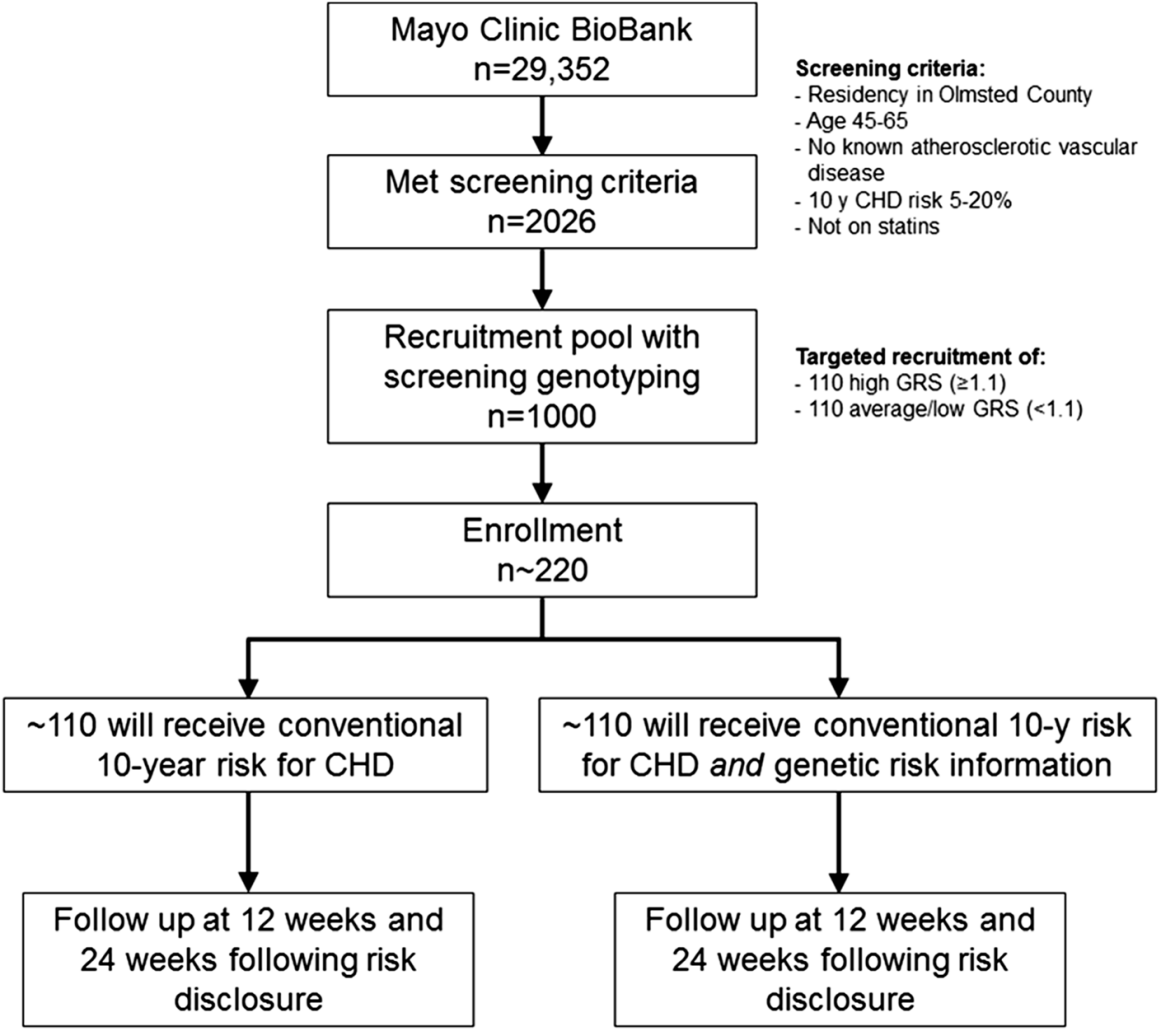
Flow Diagram of the Proposed Clinical Trial. A flow diagram illustrating the design of the MI-GENES study. A total of 2026 individuals from the Mayo BioBank met the eligibility criteria. Among those 2026, a random sample of 1000 individuals was sent for screening genotyping. A total of 968 individuals had valid screening genotyping results. Recruitment was based on screening genotyping results in order to achieve the targeted enrollment goals of ~110 individuals with high GRS (≥1.1) and ~110 with average/low GRS (<1.1) with the expectation that approximately 10-20 study participants are likely to withdraw from the study or be lost to follow-up

However, whether disclosing results of genetic testing for CHD risk influences relevant clinical outcomes is unknown. We describe the design of a randomized trial to investigate whether disclosing a GRS for CHD, based on genotypes of multiple CHD susceptibility SNPs, leads to lowering of low-density lipoprotein cholesterol (LDL-C) levels. The GRS will be incorporated into the CHD risk estimated based on a conventional risk score (CRS) [7]. We will assess whether disclosure of genetic risk for CHD affects LDL-C levels, initiation of statin therapy, changes in dietary fat intake, physical activity levels and psychosocial measures. We will test the following hypotheses: 1) in patients randomized to receive GRS, LDL-C levels at 6 months will be lower than in participants randomized to receive CRS alone; 2) participants with a ‘high’ GRS will have lower LDL-C levels at 6 months than participants in the average/low GRS category and those randomized to receive CRS alone.

A major challenge to implementing genomic medicine is integration of genomic information into the electronic health record (EHR) [8]. As part of this clinical trial, the genetic testing results will be placed in the EHR. Furthermore, we will disclose CHD risk using a decision aid integrated within the EHR to facilitate shared decision making regarding need for statin therapy [9,10]. This decision aid has been modified to include genetic risk information.

## Methods and design

### Overall study design

To maximize information yield from the study, we performed an initial screening genotyping of 28 CHD susceptibility SNPs that are not associated with blood pressure (BP) or lipid levels, in 1000 individuals from Olmsted County who were participants in the Mayo Clinic BioBank and met the eligibility criteria. We calculated GRS for each individual based on these 28 SNPs and stratified individuals into two CHD genomic risk categories: high (GRS ≥1.1), and average/low (GRS <1.1). We will enroll 110 patients in each of the two categories for the clinical trial (Fig. 1). The study coordinator will invite these patients by phone to participate in the randomized trial. He/she will inform patients about the trial, confirm eligibility, and subsequently obtain written informed consent. Specifically, the study coordinator will describe the limitations of genetic testing, the unclear medical benefit of such testing, the format for disclosure of risk, and that the results will be placed in the EHR.

Individuals who agree to participate will undergo a blood draw for genotyping of the CHD susceptibility SNPs in a Central Laboratory Improvement Amendment (CLIA)-certified laboratory. Participants will return 6-10 weeks later (Visit 2) once genotyping is completed and a GRS calculated (Table 1). At this visit, patients will be allocated (1:1) to receive GRS vs. CRS alone and will undergo a 30-min CHD risk counseling session, followed by a visit with a physician for shared decision making regarding statin use. Three months following the disclosure of CHD risk, participants will return (Visit 3) for measurement of fasting lipid levels and assessment of changes in dietary fat intake and physical activity levels. The final study visit (Visit 4) will occur three months after Visit 3. Apart from incorporating the GRS into the CRS in one arm of the study, randomized patients will receive identical exposure to CHD risk education, assessment, and consideration for means of prevention. The study was approved by the Mayo Clinic IRB and is registered at ClinicalTrials.gov (NCT01936675).

**Table 1:** Timeline for assessments

Measure	Visit 1	Visit 2	Visit 3	Visit 4
Body mass index				
Waist circumference				
Blood pressure				
Fasting lipid profile				
Fasting blood sugar				
Dietary fat intake				
Physical activity level				
Smoking status				

Visits 2, 3 and 4 occur approximately 2, 5 and 8 months after Visit 1,respectively

### Participants – eligibility requirements

Using validated electronic phenotyping algorithms, we screened 29,352 Mayo Clinic BioBank participants to identify individuals who met the following eligibility criteria: 1) residents of Olmsted County; 2) intermediate risk for CHD, i.e., a 5 %-20 % probability of adverse CHD events over the next 10 years based on the CRS; [7] 3) age 45-65 years without known CHD or CHD risk equivalent (carotid disease, peripheral arterial disease, or abdominal aortic aneurysm); 4) not on statin therapy; 5) self-identified as white ethnicity (since the GRS was derived from SNPs identified in GWAS of largely European ancestry individuals and may not be accurate for non-white individuals); 6) absence of severe comorbidity; 7) no major learning barriers, such as hearing impairment or dementia that would compromise their ability to give written informed consent.

### Screening genotyping

Of the 46 SNPs associated with CHD in GWAS, 29 are not associated with BP or lipid levels [3]. DNA from eligible Mayo Clinic BioBank participants was genotyped for 28 of the 29 CHD susceptibility SNPs on the Veracode Bead Express (Illumina®, San Diego, CA); one SNP (rs3825807) could not be genotyped for technical reasons. Genotype calls were made with Illumina’s GenomeStudio software (http://www.illumina.com), and samples with >98 % call rates across all SNPs on the array were considered for analysis. Samples with lower call rates were rerun as necessary. A GRS for each individual was calculated as previously described, taking into account the average genetic risk in the population [11]. In brief, we assumed an additive genetic model in which the genotypes are coded ‘0’ for non-risk allele homozygotes, ‘1’ for heterozygotes, and ‘2’ for risk-allele homozygotes. A weighted GRS was calculated by multiplying the logarithm of odds ratio for a particular SNP by 0, 1, or 2 according to the number of risk alleles carried by each person. We used a GRS of ≥1.1, i.e., a 10 % or greater increase in risk for CHD, to classify individuals as having ‘high’ GRS. Those with a GRS of <1.1 were classified as having average/low GRS. The “screening” GRS was performed on a research platform and was used to aid in targeted recruitment. After recruitment, GRS was re-calculated for each enrolled study participant based on repeat genotyping at a CLIA-certified laboratory.

### Recruitment and baseline characteristics

To enroll participants in the clinical trial, we will send invitation letters to a random set of patients in the high and average/low GRS categories with a goal of enrolling approximately 110 participants in each category. Assuming 5 %-10 % loss to follow up during the trial, we anticipate a final sample size of at least 200 for the study. At the initial study visit, eligibility criteria will be confirmed and informed consent obtained. We will collect information about demographic factors, educational attainment, race, marital status, medical history, social history, and family history. Participants will complete baseline surveys that assess dietary fat intake, physical activity, and psychosocial measures (Table 2). Height, weight, BP, heart rate, and waist circumference will be measured, and blood will be drawn in the fasting state to extract DNA and to measure lipids and fasting blood sugar. The 10-year probability of CHD will be assessed and calculated at the first study visit based on Wilson et al. [7].

**Table 2:** Proposed psychosocial measures

1. Numeracy and perceived risk
2. Attitude towards genetic testing
3. Genetic knowledge and understanding of genetic risk
4. Reaction to risk results and rating of test results information
5. Genetic counseling satisfaction and perceived personal control
6. Shared decision-making and physician visit satisfaction
7. Intention to change
8. Information sharing
9. Recall and decisional regret
10. Anxiety and impact of events

### CLIA Genotyping and calculation of GRS

Twenty mL of blood will be drawn and sent to a CLIA-certified laboratory and DNA will be extracted using standard procedures. All patients will undergo genotyping of the 28 CHD susceptibility SNPs using the TaqMan® procedure (Roche Molecular Diagnostics, Branchburg, NJ). A list of the 28 susceptibility SNPs and the associated genes, if known, is summarized in Table S1 of the Data Supplement. A GRS will be calculated as described above [11] and the CRS will then be multiplied by the GRS to generate an genotype-informed probability of adverse CHD events over the next 10 years (GRS).

### Outcomes

The primary outcome will be the change in LDL-C levels between visit 1 and visit 4. We will also assess whether changes in the LDL-C levels at three months following disclosure of CHD risk. We chose change in LDL-C as the primary outcome because there is a well-established causal association of LDL-C with CHD, both lifestyle changes and drug therapy can modify LDL-C levels, and reduction of LDL-C is a major therapeutic goal in both primary and secondary prevention of CHD. Behaviors related to cardiovascular health, including dietary fat intake and physical activity levels will be assessed at baseline and subsequent study visits to examine any changes over time as a result of receipt of CHD risk information. We will assess several psychosocial measures listed in Table 2. The use of validated measures and selection of instruments previously reported in the genetic literature will enable comparison of the current findings with existing literature [12].

### Sample size and power

In general, studies have confirmed a 5 to 15 % decrease in LDL-C with diet and lifestyle changes and a 30 to 40 % decrease in LDL-C with statin therapy [13,14]. We aimed to detect an LDL-C change of 10 mg/dL to test the first hypothesis that patients randomized to receive GRS would have lower LDL-C levels than patients randomized to CRS alone. For the second hypothesis which will test whether high GRS participants would have lower LDL-C levels that those with low/average GRS and those randomized to receive CRS alone, we aimed to detect an LDL-C change of 15 mg/dL. We assumed the standard deviation of LDL-C change in the entire group to be 25 mg/dL. Under this assumption, the detectable 2-group difference for this change (with 80 % power and 5 % Type I error rate) [hypothesis 1] is 25*sqrt(2/100)*2.8 = 9.9 mg/dL. For hypothesis 2, the detectable decreases in high GRS versus average/low GRS and high GRS versus CRS (with 80 % power and 5 % Type I error rate) are 25*sqrt(2/50)*2.8 = 14 mg/dL and 25*sqrt(1/50 + 1/100)*2.8 = 12.1 mg/dL. Thus, the trial is well-powered to identify an effect of genetic testing that is in the same range as that of diet and exercise.

### Randomization

The second study visit will be scheduled 6-10 weeks after the initial visit to allow for completion of genotyping and calculation of GRS. One of the study staff will allocate patients (1:1) to the two arms, one group receiving genetically informed 10-year CHD risk (GRS) and the other conventional risk factor information alone (CRS). Randomization will be performed by means of a computer-generated random sequence with stratification for age, gender, and positive family history for CHD using the method previously described by Pocock and Simon [15].

### Disclosure of CHD risk

The CHD risk estimate will be disclosed by the genetic counselor during a 30-min semi-scripted session. Patients randomized to GRS will be shown a pictograph that incorporates the revised 10-year CHD risk based on the genotypes of the 28 CHD susceptibility SNPs. The control group will be shown a pictograph based on the CRS. The pictograph depicts 100 people “like the participant” and indicates how many in the next 10 years could be expected to experience an adverse CHD event and how many will not. The genetic counselor will help participants interpret and understand their results, highlighting the probabilistic nature of the genetic testing and that lifestyle factors such as diet, exercise, and smoking are major risk factors for developing CHD. The counselor will encourage participants to sign an action plan for behavioral change that includes increased physical activity and reduced dietary fat intake and smoking cessation if the participant is a smoker. Participants will be provided with a *Frequently Asked Questions* sheet that reiterates the key points conveyed by the genetic counselor at the visit.

### Shared decision making regarding need for statin therapy

Following the visit with the genetic counselor, the patients will see a physician in the preventive cardiology clinic. The physicians will receive training in the use of the decision aid that has been modified to incorporate the genotype-informed estimate of CHD risk. A mock session will be conducted to increase familiarity with the tool. During the patient-physician encounter the focus will be on shared decision making regarding the need for statin therapy. Following this visit, patients will be asked to fill in a questionnaire assessing satisfaction with the physician visit and the shared decision-making process. We will videotape all genetic counselor and physician encounters and assess the fidelity of the genetic counselor and physicians to instructions on the use of the statin choice decision aid [16]. We will use the OPTION instrument [17] to quantify the degree to which physicians involved study participants in the decision making process related to statin therapy. We will also perform qualitative analyses of the videotaped encounters to assess how patients react to genomic results.

### Assessment of changes in psychosocial measures

The participants’ comprehension and perception of CHD risk, their response to genetic risk results, and how they share such information will be assessed by specific surveys. We will measure perceived personal control and decisional regret to better understand ways in which disclosure of genetic testing results affects the individual patient. Such measures will provide novel perspectives on the delivery and receipt of information that will be relevant to counseling and informed consent practice.

### Follow-up

Three and six months after disclosure of CHD risk, participants will return to fill out study questionnaires and undergo a blood draw for fasting plasma lipid levels. At the 6-month visit, participants in the CRS arm will also receive their GRS and will be instructed to discuss their results with their primary care providers. We will review the medical records 6 and 12 months after disclosure of GRS to study participants originally assigned to receive CRS only earlier in the study, to assess whether disclosure of a genotype-informed CHD risk leads to initiation of statin therapy or ordering of additional tests.

### Statistical methods

All survey data will be exported from REDCap (Research Electronic Data Capture) [18] to a SAS database for analyses. Data analysts will be blinded to allocation. Descriptive data will be provided for all measures. The frequency (%) of categorical factors will be compared using either the Chi-Square or Fisher’s exact test and t-tests for continuous outcomes such as LDL-C levels. The primary groups analyzed will be the randomized treatment groups – CRS and GRS. We will also conduct supplementary analyses comparing 3 groups: CRS, high GRS, and average/low GRS.

The use of validated, standard measures for many of the behavioral and psychosocial measures will enable comparison of the data with other studies. Within-participant analyses (using each participant as his/her own control) will be performed to evaluate changes in these measures over time. Difference in scores will be examined for the outcomes by comparing post-results assessments to baseline assessments. Logistic regression will be used for binary outcomes (e.g., % achieve accurate understanding of their CHD risk) to compare the change by arm with odds ratios and their associated 95 % confidence intervals. Adjusted comparisons of binary and continuous outcomes will be performed using regression models. All secondary analyses will be based on two-sided tests at a significance level of 0.05. We will conduct subgroup analyses to assess interactions between the intervention and: a) sex and b) family history of CHD.

## Results

Of 29,352 individuals enrolled in the Mayo Clinic BioBank, we identified 2026 individuals who met the eligibility criteria for the study. Of these 2026 individuals, a random subset of 1000 was genotyped for the 28 SNPs associated with CHD independent of blood pressure and lipid levels to calculate a GRS for each individual. After quality control checks, genotyping results were valid for 968 individuals who became the recruitment pool for this study. Characteristics of the recruitment pool are shown in Table 3. The mean age of the source population was 57 years, 55 % were women, and the mean 10-year probability of CHD was ~8 %. The mean GRS in the high-risk group was 1.33, and the mean GRS in the non-high-risk group was 0.85. There was no significant correlation between GRS and CRS (Pearson correlation coefficient = 0.029, *P* = 0.38), in the overall sample (n = 968).

**Table 3:** Characteristics of Mayo Clinic BioBank patients who met the eligibility criteria

	Overall	GRS ≥1.10	GRS <1.10
n	968	311	657
Age, years	57.6 ± 5.41	57.6 ± 5.37	57.5 ± 5.43
Women	531 (55 %)	169 (54 %)	362 (55 %)
CRS, %	7.98 ± 3.16	7.89 ± 3.13	8.02 ± 3.18
GRS	1.00 ± 0.28	1.33 ± 0.20	0.85 ± 0.16

Continuous variables are represented as mean ± standard deviation and categorical variables as n (%)

## Discussion

Of the multiple CHD susceptibility genetic variants discovered by GWAS the majority are not associated with conventional risk factors. Genotyping such variants is a potential means of refining risk stratification for CHD [1,19,20] and therefore has major public health implications as CHD remains the leading cause of death in much of the world and often the first manifestation is sudden death or myocardial infarction. Although the odds ratios/relative risks associated with common genetic variants are modest, aggregating information from multiple genetic markers may provide additive information for risk stratification. Our goal in this study will be to explore whether disclosure of genetic risk of CHD influences LDL-C levels, behavioral changes (dietary fat intake and physical activity) as well as shared decision making regarding statin therapy.

We chose to include individuals at intermediate risk for CHD as decisions regarding statin initiation are often complex and motivating patients to change diet and lifestyle can be challenging. Targeting these individuals with preventive measures to reduce long-term risk could result in substantial public health benefit [21,22]. The design of our proposed study differs significantly from a previously proposed trial design for disclosing CHD genetic risk information [23]. We performed a screening genotyping initially to identify and enroll individuals in different categories of GRS for CHD. Furthermore, blood draw and genotyping were done in a CLIA-environment and results placed in the EHR. Additional outcomes of interest in our study include changes in lifestyle and psychosocial measures.

Our primary outcome in this study will be change in LDL-C levels following disclosure of CHD risk. LDL-C is a causal risk factor for CHD and both pharmacologic as well as lifestyle interventions can lower LDL-C. Furthermore, standardized assays are readily available to measure lipid levels and estimate LDL-C. We will assess whether any changes in LDL-C levels are due to lifestyle changes, pharmacological interventions, or both. We will obtain information about diet, physical activity, numeracy, perception of genetic risk, anxiety, intention to change and sharing of test results.

Although it is well known that low dietary fat and increased physical activity reduce the risk of CHD, motivating patients to adopt and sustain lifestyle changes is often difficult. Knowledge of genetic risk may improve patient outcomes by increasing patient motivation (e.g., people who recognize and accept their high genetic risk are more motivated to mitigate it). There is also the possibility that for individuals at relatively low genetic risk, motivation for lifestyle changes may be reduced. We will therefore explore whether disclosing personal genetic risk information for CHD can affect motivation for lifestyle changes.

Recent guidelines [9] emphasize the need for shared decision making when considering the use of statin medications for lowering CHD risk. We modified an existing statin-choice decision aid to incorporate genetic risk information for use in the trial. The decision aid includes pictographs to disclose the 10-year probability of having a CHD event as well as the impact of statin use on such events. Such visual depictions help patients as well as physicians [24] to better comprehend statistical probabilities related to risk of disease. Our decision aid was designed to facilitate shared decision making in the setting of disclosure of CHD genetic risk and we plan to explore in depth how decision making takes place based on quantitative and qualitative analyses of video recordings of the patient-genetic counselor and the patient-MD encounters.

The number of patients undergoing genetic testing is increasing, motivating the study of not only medical outcomes but also the impact of disclosure of genetic risk on psychosocial measures. We have included measures of perceived personal control and decisional regret to better understand ways in which disclosure of genetic testing results affects the individual patient. We will assess participants’ understanding of genomic results and their views about placing genomic data in the EHR, sharing genomic results with family members and others, and any regrets about pursuing genomic testing (Table 2). As a result we will be able to obtain novel perspectives on the delivery and receipt of genetic risk information that will be relevant to counseling patients about genetic testing for common diseases.

Several limitations of our proposed clinical trial need to be mentioned. Study participants were drawn from people who enrolled in the Mayo Clinic BioBank, and these individuals may not be truly representative of the community at large. However, this approach allowed us to recruit individuals in a targeted fashion based on the genetic risk score. Having a genetic counselor disclose CHD risk may not be feasible in the busy clinical flow of daily practice. However, web-based tools, such as the one we have developed, could be used by other care providers (e.g., a nurse) for disclosing genetic risk. Additional studies will be needed to study diverse ethnic groups and ‘real world’ settings.

In conclusion, we describe the design for the Myocardial Infarction GENES (MI-GENES) study which is among the first clinical trials to assess changes in a health-related outcome after disclosure of genetic risk for CHD in the clinical setting. The proposed trial will provide insights into the clinical utility of genetic testing for CHD risk assessment.

### Trial status

This clinical trial is still ongoing. Final follow-up for all study participants and assessment of end-points will be completed by December 2015.

## Additional file

Additional file 1: Table S1. Genetic Loci Associated with Coronary Heart Disease Used in Genetic Risk Score Calculation. (PDF 154 kb)

## Abbreviations

CHD: Coronary heart disease
CLIA: Central laboratory improvement amendment
CRS: Conventional risk score
GRS: Genetic risk score
GWAS: Genome-wide association study
LDL-C: Low lipoprotein density cholesterol
MI-GENES: Myocardial Infarction GENES study
SNP: Single-nucleotide polymorphism
