# Supplementary material for: Design of a randomized controlled trial of disclosing genomic risk of coronary heart disease: the Myocardial Infarction Genes (MI-GENES) study
Source: BMC Med Genomics. 2015 Aug 15;8:51. doi: 10.1186/s12920-015-0122-0 (PMC4536729; doi:10.1186/s12920-015-0122-0)
Supplement: Additional file 1: Table S1. — Genetic Loci Associated with Coronary Heart Disease Used in Genetic Risk Score Calculation. (PDF 154 kb) [file 12920_2015_122_MOESM1_ESM.pdf]

SUPPLEMENTAL MATERIAL

Supplementary Table 1. ***Genetic Loci Associated with Coronary Heart Disease Used in Genetic Risk Score Calculation***

Gene	SNP	CHR	Risk Allele	Risk Allele OR
MIA3	rs17465637	1	C	1.14
PPAP2B	rs17114036	1	A	1.11
IL6R	rs4845625	1	T	1.04
WDR12	rs6725887	2	C	1.12
ZEB2-AC074093.1	rs2252641	2	G	1.04
VAMP5-VAMP8-GGCX	rs1561198	2	A	1.05
MRAS	rs9818870	3	T	1.07
EDNRA	rs1878406	4	T	1.06
SLC22A4-SLC22A5	rs273909	5	C	1.09
TCF21	rs12190287	6	C	1.07
PHACTR1	rs9369640	6	A	1.09
KCNK5	rs10947789	6	T	1.06
PLG	rs4252120	6	T	1.06
ANKS1A	rs17609940	6	G	1.07
7q22 *BCAP29*	rs10953541	7	C	1.08
HDAC9	rs2023938	7	G	1.07
CDKN2BAS1	rs1333049	9	C	1.23
CXCL12	rs2047009	10	C	1.05
KIAA1462	rs2505083	10	C	1.06
PDGFD	rs974819	11	A	1.07
COL4A1-COL4A2	rs4773144	13	G	1.07
COL4A1-COL4A2	[†]rs9515203	13	T	1.08
FLT1	rs9319428	13	A	1.05
HHIPL1	rs2895811	14	C	1.06
RAI1-PEMT-RASD1	rs12936587	17	G	1.06
SMG6	rs216172	17	C	1.07
UBE2Z	rs46522	17	T	1.06
Gene desert (*KCNE2*)	rs9982601	21	T	1.13

CHD: coronary artery disease; CHR: Chromosome; OR: odds ratio
